# Wide en-bloc thymectomy and venous axis reconstruction in Masaoka stage IIIB thymoma: a case report and literature review

**DOI:** 10.1093/jscr/rjae263

**Published:** 2024-05-02

**Authors:** Mohamad A Nahas, Raghad Samha, Mohamad Shbat, Sawsane A Ghaddar, Afnan W M Jobran, Layal Msheik, Zaher Al nahhas, Hussain Chaban

**Affiliations:** Division of Vascular and Endovascular Surgery, AlAssad Damascus University Hospital, Faculty of Medicine, Damascus University, Damascus, Syria; Faculty of Medicine, AlBaath University, Homs, Syrian Arab Republic; Department of Thoracic Surgery, Al-Assad university Hospital, Damascus, Syria; Faculty of Medical Sciences, Lebanese University, Beirut, Lebanon; Faculty of Medicine, Alquds University, Jerusalem, Palestine; Faculty of Medical Sciences, Lebanese University, Beirut, Lebanon; Department of Radiology, Damascus hospital, Damascus, Syrian Arab Republic; Department of Thoracic Surgery, Al-Assad university Hospital, Faculty of Medicine, Damascus University, Damascus, Syria

**Keywords:** thymoma, SVC, brachiocephalic, bypass, case report

## Abstract

Thymomas are rare tumors originating from thymic tissue and rarely metastasize. They can be diagnosed either incidentally or symptomatically when compressing or invading nearby structure. A 36-year-old man presented with significant high-grade fever, chest pain that worsens upon lying down, and dyspnea. A chest X-Ray and computed tomography followed by biopsy confirmed the diagnosis of thymoma. The management included chemotherapy cycles, followed by surgery. Pericardiectomy was performed with en-bloc thymectomy and partial resection of the infiltrating lung. Venous drainage was restored by 8/16 mm inverted bifurcated brachiocephalic-superior vena cava Dacron bypass. The pericardium was reconstructed by a synthetic Dacron patch, and the right diaphragm metastasis was resected. Neoadjuvant chemotherapy was initiated. After 3 months of follow-up, no recurrence was evidenced by computed tomography.

## Introduction

Thymomas are tumors arising from the thymic tissue and they are the most prevalent neoplasms of the anterior mediastinum [1]. Thymoma is rare, with a low incidence of 0.13 to 0.32/100 000/year, accounting for just 0.2 to 1.5% of all malignancies [2].

Research has discovered a strong link between thymomas and paraneoplastic diseases mainly myasthenia gravis [3]. It can be diagnosed as an accidental discovery on imaging or because of symptoms associated with mass effect or paraneoplastic disease [4]. Computed tomography (CT) scan and fine needle aspiration are the main diagnostic tools [1].

Differential diagnosis includes lymphoblastic lymphoma, thymic cancer, thyroid carcinoma, and thymic hyperplasia [5].

Thymomas are mostly benign and encapsulated, but up to 30% of them cross the capsule and infiltrate adjacent tissues [6]. Distant metastases via regional lymph nodes are infrequent [7].

Treatment involves surgical resection for encapsulated and invasive thymomas, followed by postoperative radiotherapy for invasive ones.

Herein, we presented a case of invasive thymoma that infiltrated both right and left brachiocephalic veins and superior vena cava (SVC), which was managed successfully by en-bloc resection and inverted 8/16 mm Dacron brachiocephalic-SVC bypass.

## Case presentation

A 36-year-old male was admitted to the thoracic surgery department complaining of severe chest pain that worsens upon lying down, accompanied by dyspnea, and a high-grade fever with delirium. Medical and family history were unremarkable, only declared 40-pack-year smoking history.

Chest X-ray evidenced a mediastinal mass. A contrast-enhanced computed tomography and CT-guided biopsy suggested the diagnosis of thymoma, which was confirmed by immunohistochemical staining with positive CK, Tdt, and CD5. Chemotherapy was recommended before surgery to reduce tumor size. Twenty chemotherapy cycles were planned over two years, the patient underwent six cycles; the tumor shrank however, the patient discontinued the therapy when symptoms subsided, as a result, the tumor recurred and no longer responded to the other 14 chemotherapy cycles.

**Figure 1:**
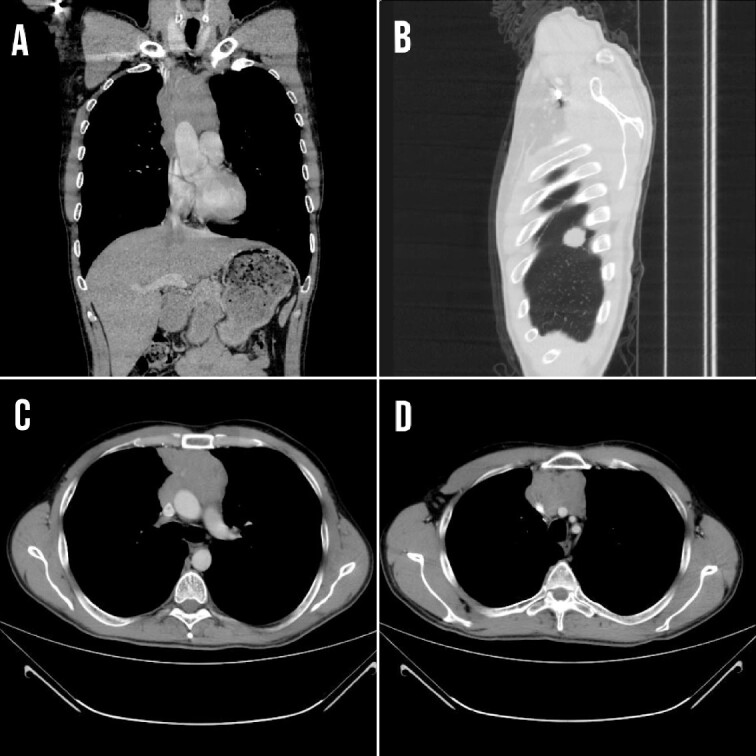
(A) CT coronal section showing the mediastinal lesion. (B) Right diaphragm metastasis at the posterior costal margin at the level of 4 and 5 thoracic ribs (C and D) Transverse section demonstrating the mass that compressed and invaded the major neck veins and the SVC.

The symptoms persisted and aggravated with dysphagia, weight loss, dyspnea on exertion, and facial and upper limb edema due to compression of the SVC. Laboratory test results are demonstrated in Table 1. A CT scan showed that the mass compresses and invades the major neck veins and the SVC with a thrombus inside, with the presence of a right diaphragmatic metastasis (Fig. 1).

**Table 1:** Laboratory test results.

**Investigation**	**Patient test results**	**Reference value range**
Neutrophils	39.2	40–70%
Monocytes	13.7	2–6%
Basophils	0.9	1–2%
Red blood cells (RBC)	3.88	4.5–5.5 cells/mcL
Hemoglobin (Hb)	11.9	13–16 g/dL
Hematocrit (Hct)	37.4	38–53%
Mean corpuscular volume (MCV)	96.4	82–96 fl
Red cell distribution width (RDW)	18.4	11.5–14.5%

**Figure 2:**
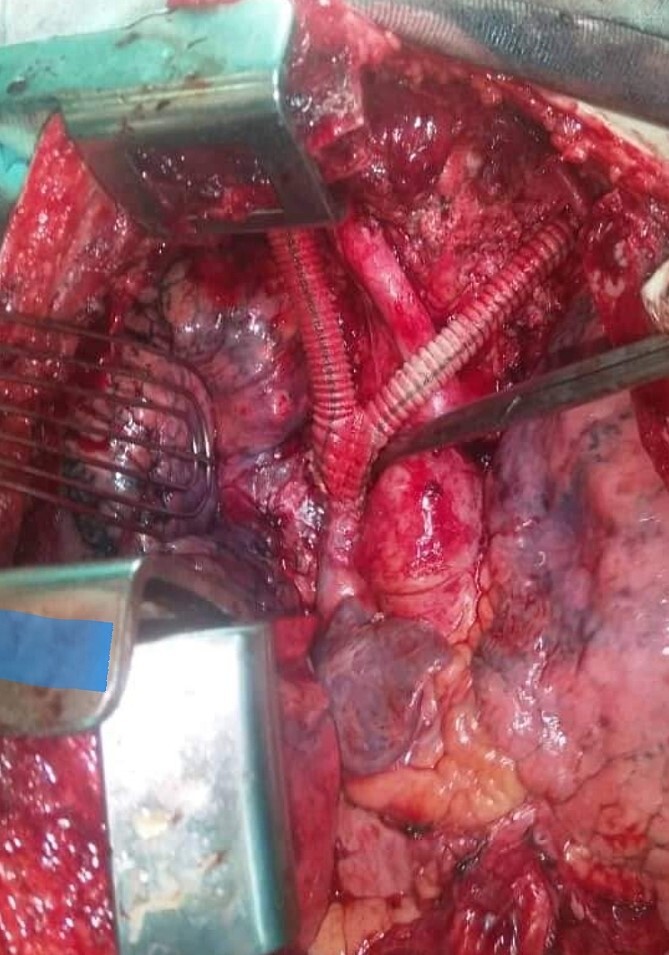
The inverted bifurcated 8/16 mm Dacron bypass between both right and left brachiocephalic veins and the superior vena cava.

Surgery was decided. A median sternotomy was performed. A noteworthy mass infiltrating both right and left brachiocephalic veins, the SVC, and invading the right lung parenchyma and the pericardium was found. Pericardiectomy was performed with en-bloc thymectomy, preserving the right and left phrenic nerves with partial resection of the infiltrating lung. Venous drainage was restored by 8/16 mm inverted bifurcated brachiocephalic-SVC Dacron bypass (Fig. 2), the pericardium was reconstructed by a synthetic Dacron patch, and the right diaphragm metastasis was resected (Video 1).

**Figure 3:**
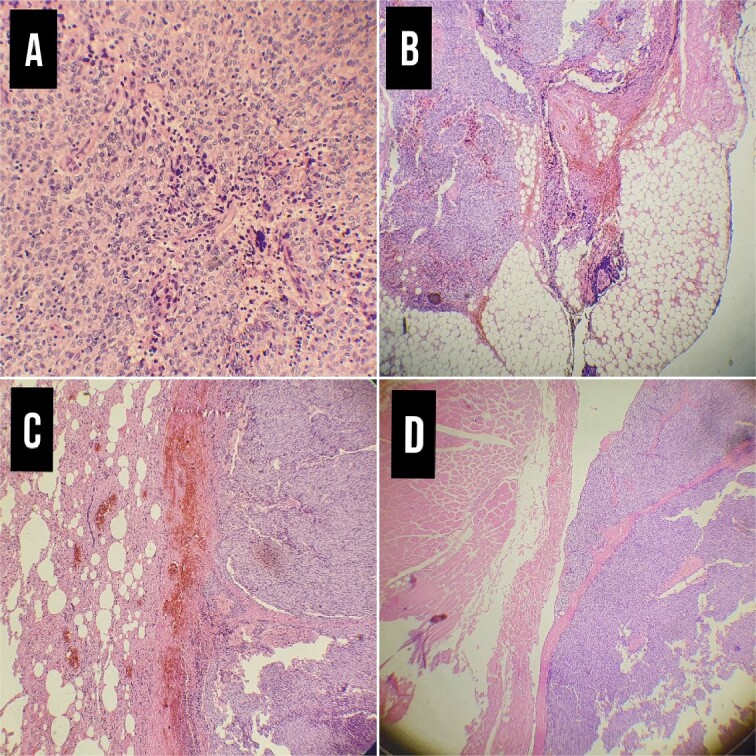
Pathological examination. (A) Thymoma WHO type 3B × 20, (B) Infiltration of the tumor in the adipose tissue of the pleura ×4. (C) Infiltration of the tumor in lung tissue**×** 4**.** (D) Infiltration of the tumor in the chest wall muscles.

The histopathological examination result was a Masaoka type IIIB thymoma, which measures (12 cm). Lymph vascular invasion presented along with necrosis, and surgical margins were free of tumor, with pathologic staging of pT3, pNx, and pM1 (Fig. 3).

After surgery, enoxaparin sodium 60 mg injections were given twice a day for a week along with ceftriaxone. The patient was discharged with a direct oral anticoagulants regimen after 1 week, and neoadjuvant chemotherapy was started.

A 3 months follow-up evidenced no significant complications, and the patient was stable; the CT scan showed no recurrence.

## Discussion

Thymoma, originating from the thymic epithelium, is the most common tumor of the anterior mediastinum [8, 9], They occur with similar frequency in both genders, typically affecting individuals in their fifth and sixth decades [8] contrary to our case, in which the patient was relatively young. While invasive thymomas frequently infiltrate proximal structures such as veins and arteries, intraluminal venous invasion is an exceedingly rare phenomenon [9, 10].

Thymomas are frequently discovered incidentally, with symptoms attributable to compression emerging in about half the cases [8, 11]. Common presenting symptoms include cough, dyspnea, chest pain, myasthenia gravis symptoms, and SVC syndrome [8, 12, 13]. SVC syndrome is due to extrinsic compression by a tumor or, very rarely, intrinsic thrombosis. Determining etiology necessitates advanced imaging modalities such as CT, MRI, echocardiography, or venography. Management strategies hinge on the underlying cause, encompassing surgical resection, thrombolysis, percutaneous angioplasty, and anticoagulation [14]. In our case, the thymoma extensively invaded the SVC and brachiocephalic veins, leading to SVC syndrome.

Effective management of thymomas typically involves chemotherapy and radiotherapy; however, complete en bloc resection remains the treatment of choice [8, 10]. Radiotherapy is generally for incompletely resected tumors, while chemotherapy is for metastasis [8]. In the context of SVC syndrome, surgery is a crucial intervention to restore venous return, given the invasive growth pattern of thymomas [9]. In such cases, combined atrial and venous wall resections are usually performed to address the extensive involvement of these tumors [10]. In our presented case, surgical En-bloc total resection of the mass was done, excision resection of metastases to the diaphragm, pericardium, and lung, and the venous drainage was restored by an inverted brachiocephalic- superior vena cava 8/16 mm Dacron bifurcated bypass.

Thymoma management should be tailored to suit the unique presentations of individual patients, as presented in the review table (Table 2), with a primary emphasis on achieving complete resection [8]. For an aggressive thymic clear cell carcinoma, successful treatment included complete resection, encompassing thymothymectomy, cervicomediastinal nodal dissection, and right upper lobectomy with hilar lymphadenectomy [15]. In the context of multiple thymomas, an extensive thymectomy was recommended in three cases to prevent recurrence [16]. Notably, in a case of advanced thymic carcinoma, Lenvatinib was administered for 6 months, resulting in a partial response marked by a 30% reduction in tumor size, decreased mediastinal lymph nodes, and resolution of pericardial effusion, emphasizing the importance of investigating Lenvatinib’s impact on thymomas [17]. In addition to these treatment strategies, the use of graft bypass in the case of SVC syndrome can play a pivotal role in ensuring successful outcomes, as presented in our case.

**Table 2:** Literature review of thymoma management.

References	Gender/ Age	Main Complain	Metastasis	Invasions	Management	Follow up
[14]	M/78	Acute left arm swelling	Left IV	Extended deep into the mediastinum	The mass was removed through a right atriotomy, a Fogarty catheter was used to remove as much as possible of the tumor material and thrombus, A single vein bypass graft was placed to the LAD coronary artery, then radiotherapy	NED
[15]	M/42	Asymptomatic	Marked nodal metastases involved right hilar, mediastinal, and left supraclavicular regions.	None	- Complete resection including thymothymectomy, cervicomediastinal nodal dissection, and right upper lobectomy with hilar lymphadenectomy - Postoperative chemoradiation therapy	Brain metastasis occurred 1 year after surgery.
[16]	C1, M/69 C2, M/74 C3, M/46	Asymptomatic	Multiple thymomas develop from identical tumor genesis events in each patient, thus resulting in the similar histological findings.	C1-microscopic invasion into the surrounding thymic tissue. C2- invasion of the tumor into the right middle lobe of the lung. C3- microscopic invasion into the surrounding thymic tissue.	C1- Thymo-thymomectomy C2- Thymo-thymomectomy and post-operative radiotherapy C3- Thymo-thymomectomy	C1- NED C2- NED C3- NED
[17]	M/50	Anterior chest discomfort	Left mediastinal lymph node metastases	- Left upper lobe of the lung	Lenvatinib therapy was started then Complete salvage resection was done with radical thymectomy.	NED
[18]	M/65	Non-productive cough	None	- Right upper lobe of the lung - right BCV - left BCV - SVC	Chemotherapy Then median sternotomy, the right upper lobe was initially mobilized by dividing the upper lobe pulmonary arteries/veins and bronchus. The great veins including the SVC/RA junction proximal and distal to the tumor were then dissected for vascular control. Then radiation	NED
[19]	C2, M/59	C2- Ptosis palpebrae, Facial edema	None	- SVC - RA	Thymectomy with SVC-RA tumor resection and venous resection of the SCV and the BCV, bilateral BCV-RA bypass with PTFE (10 mm), followed by radiation and chemotherapy	Lung metastasis after 4 years
[20]	F/79	Asymptomatic	Intrapulmonary metastasis	None	VATS thymectomy	NED
[21]	M/9	Community-acquired pneumonia.	None	- The left upper lung lobe - phrenic nerve - pericardium - hypertrophied mediastinal lymph nodes	Left upper lobectomy and resection of the mediastinal mass and lymph nodes	NED
[22]	M/56	SVC syndrome	None	- pericardium - SVC	The tumor in the right atrium was resected from the SVC by blunt digital dissection, and was catheterized by Fogarty catheter, then radiotherapy	NED
[23]	M/68	Hemoptysis	None	- SVC - RA	The tumor and left brachiocephalic vein were resected, and the tumors in the SVC and RA were removed then chemotherapy	NED
[24]	M/49	Multiple left pleuropericardial thickenings	None	Left upper mediastinal invasion	Macroscopically complete tumor resection was achieved via subtotal pleurectomy, two pulmonary wedge resections and partial pericardiectomy, then chemotherapy	NED
[25]	M-59		Lymph node metastasis.	- Pericardial invasion	Median sternotomy with mediastinal mass resection and anterior pericardial sac and mediastinal aortopulmonary window LN resection, followed by radiation	NED

Abbreviations list: M: male; F: female; NED: no evidence of disease; C: case; LN: lymph node; SVC: superior vena cava; RA: right atrium; VATS: video-assisted thoracoscopic surgery; BCV: brachiocephalic vein; IV: innominate vein

## Conclusion

We recommend considering the inverted graft bypass in this atypical location to be done, such bypass was performed between both brachiocephalic veins and SVC, with an unexpectedly excellent post-surgical recovery. 

## Supplementary Material

video_1_rjae263

## Data Availability

Not applicable. All data (of the patient) generated during this study are included in this published article and its supplementary information files.
